# A Decrease in Maternal Iron Levels Is the Predominant Factor Suppressing Hepcidin during Pregnancy in Mice

**DOI:** 10.3390/ijms241814379

**Published:** 2023-09-21

**Authors:** Sheridan L. Helman, Sarah J. Wilkins, Jennifer C. J. Chan, Gunter Hartel, Daniel F. Wallace, Gregory J. Anderson, David M. Frazer

**Affiliations:** 1 Molecular Nutrition Laboratory, QIMR Berghofer Medical Research Institute, Herston, QLD 4006, Australia; sheridan.helman@qimrberghofer.edu.au (S.L.H.); s213038@gmail.com (J.C.J.C.); 2 Iron Metabolism Laboratory, QIMR Berghofer Medical Research Institute, Herston, QLD 4006, Australiagreg.anderson@qimrberghofer.edu.au (G.J.A.); 3 Statistics Unit, QIMR Berghofer Medical Research Institute, Herston, QLD 4006, Australia; gunter.hartel@qimrberghofer.edu.au; 4 School of Public Health, The University of Queensland, Herston, QLD 4006, Australia; 5 School of Nursing, Queensland University of Technology, Kelvin Grove, QLD 4059, Australia; 6 School of Biomedical Sciences and Centre for Genomics and Personalised Health, Queensland University of Technology, Kelvin Grove, Brisbane, QLD 4059, Australia; d5.wallace@qut.edu.au; 7 School of Biomedical Sciences, Queensland University of Technology, Gardens Point, QLD 4000, Australia; 8 School of Biomedical Sciences, The University of Queensland, St. Lucia, QLD 4067, Australia

**Keywords:** pregnancy, iron homeostasis, hepcidin

## Abstract

In order to supply adequate iron during pregnancy, the levels of the iron regulatory hormone hepcidin in the maternal circulation are suppressed, thereby increasing dietary iron absorption and storage iron release. Whether this decrease in maternal hepcidin is caused by changes in factors known to regulate hepcidin expression, or by other unidentified pregnancy factors, is not known. To investigate this, we examined iron parameters during pregnancy in mice. We observed that hepatic iron stores and transferrin saturation, both established regulators of hepcidin production, were decreased in mid and late pregnancy in normal and iron loaded dams, indicating an increase in iron utilization. This can be explained by a significant increase in maternal erythropoiesis, a known suppressor of hepcidin production, by mid-pregnancy, as indicated by an elevation in circulating erythropoietin and an increase in spleen size and splenic iron uptake. Iron utilization increased further in late pregnancy due to elevated fetal iron demand. By increasing maternal iron levels in late gestation, we were able to stimulate the expression of the gene encoding hepcidin, suggesting that the iron status of the mother is the predominant factor influencing hepcidin levels during pregnancy. Our data indicate that pregnancy-induced hepcidin suppression likely occurs because of reductions in maternal iron reserves due to increased iron requirements, which predominantly reflect stimulated erythropoiesis in mid-gestation and increased fetal iron requirements in late gestation, and that there is no need to invoke other factors, including novel pregnancy factor(s), to explain these changes.

## 1. Introduction

Iron deficiency and its more severe manifestation, iron deficiency anemia, are common in pregnant women due to the high iron demands of the developing fetus and are associated with adverse pregnancy outcomes such as increased maternal morbidity and mortality, preterm birth, intrauterine growth restriction and impaired cognitive development in the infant [1,2,3]. To reduce the risk of iron deficiency, pregnant mammals suppress the production of the peptide hormone hepcidin, which is often regarded as the master regulator of iron homeostasis [4,5]. This 25-amino acid peptide is produced by the liver and secreted into the bloodstream in response to changes in body iron demand [6]. Circulating hepcidin maintains iron homeostasis by binding to ferroportin, which is expressed predominantly on the surface of cells that actively export iron such as intestinal enterocytes, hepatocytes and macrophages [7,8,9]. Ferroportin is the only known mammalian iron export molecule and the binding of hepcidin induces the internalization and degradation of the ferroportin/hepcidin complex [10], reducing iron efflux. The net effect of decreasing hepcidin production during pregnancy is an increase in dietary iron absorption and the release of iron from storage sites in the liver and reticuloendothelial system. However, the factors causing hepcidin suppression during pregnancy have not been determined.

Under normal physiological conditions, hepcidin is regulated by iron parameters such as the amount of iron stored in the liver, the iron demands of the erythroid marrow and circulating iron bound to transferrin [11,12,13]. These parameters are also altered during pregnancy, with erythropoiesis elevated to accommodate the expanding maternal blood volume [14,15] and the iron requirements of the developing fetus increasing as pregnancy progresses [14,15], both of which lead to a decrease in maternal liver and serum iron levels. It is, therefore, feasible that these changes in maternal iron status are solely responsible for hepcidin suppression, particularly during the latter stages of pregnancy. However, previous studies in pregnant mice showed that hepcidin is suppressed in mid-gestation, when the iron requirements of the developing embryos are minimal [16,17], and that hepcidin suppression is retained when hepatic iron stores are elevated, as demonstrated in mice injected with iron dextran at conception [16]. These data have led to suggestions that a novel pregnancy factor regulates maternal hepcidin expression, independent of iron status [16,18,19,20]. However, even in the iron-loaded mice mentioned above, iron levels were relatively reduced in pregnancy [16], suggesting that maternal iron levels could play a role in hepcidin regulation at this time.

In the current study, we utilize the pregnant mouse as a model organism to conduct an analysis of the effect of maternal iron status on hepcidin expression, as this has not been examined in detail. By examining the utilization of iron during pregnancy and by manipulating iron levels in pregnant mice, we provide evidence that increased iron requirements are the predominant influence on hepcidin levels during pregnancy.

## 2. Results

### 2.1. Expression of the Gene Encoding Hepcidin in Pregnant Dams Reflects Maternal Iron Status at E12.5 and E18.5

**Figure 1:**
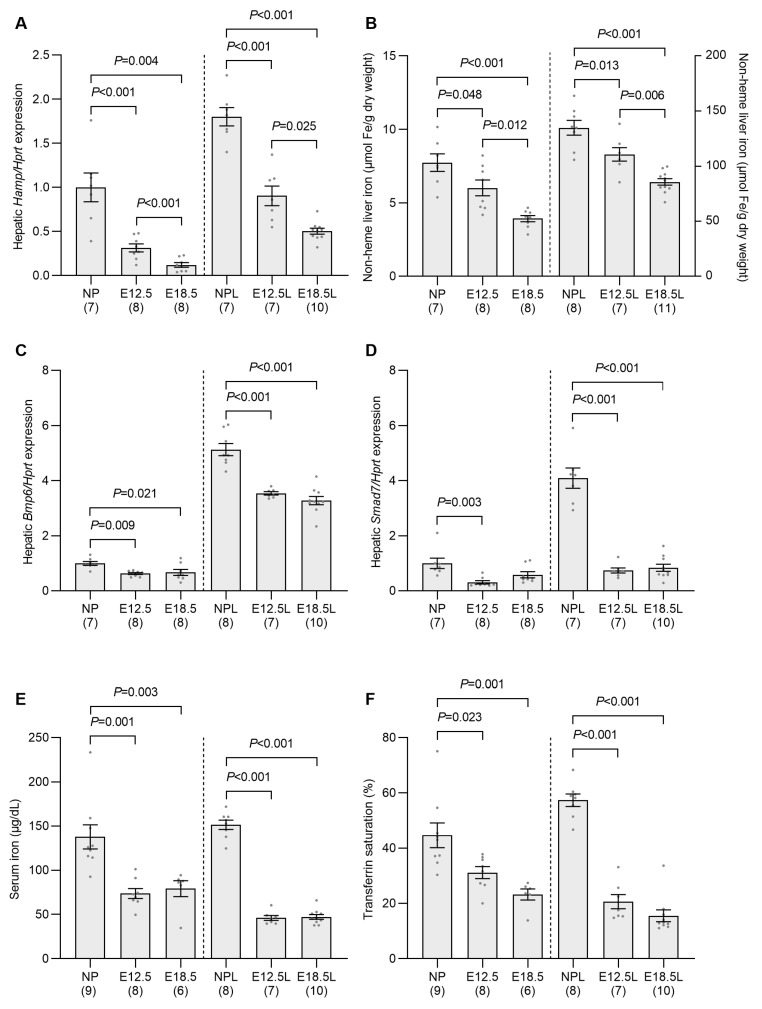
Hepatic *Hamp* expression is suppressed in pregnancy and correlates with reduced transferrin saturation and iron stores. Non-iron-loaded and iron-loaded pregnant mice were examined at E12.5 or E18.5 and compared to age-matched nonpregnant mice to investigate the effect of pregnancy progression on hepatic *Hamp* expression (**A**), nonheme liver iron (**B**), hepatic *Bmp6* (**C**) and *Smad7* expression (**D**), serum iron (**E**) and transferrin saturation (**F**). Gene expression was calculated relative to the housekeeping gene *Hprt* and is expressed as a proportion of levels in the nonpregnant group. Data are expressed as mean ± SEM with the number of mice in each group indicated in parentheses along the *x*-axis. Statistically significant differences between groups were determined using one-way ANOVA followed by either Tukey or Games–Howell post-hoc testing, with *p* values indicated for each significantly different comparison. NP: nonpregnant mice; E12.5: pregnant mice studied at embryonic day 12.5; E18.5: pregnant mice studied at embryonic day 18.5; NPL: iron-loaded nonpregnant mice; E12.5L: iron-loaded pregnant mice studied at embryonic day 12.5; E18.5L: iron-loaded pregnant mice studied at embryonic day 18.5.

To determine the effect of pregnancy on maternal iron status and hepcidin antimicrobial peptide (*Hamp*) expression (the gene encoding hepcidin), we examined both non-iron-loaded and iron-loaded pregnant mice at mid-gestation (E12.5) and late gestation (E18.5), using nonpregnant mice as controls. In agreement with previously published data, hepatic *Hamp* mRNA was significantly reduced at both E12.5 and E18.5 when compared with nonpregnant controls, with E18.5 levels also significantly lower than at E12.5, in both non-iron-loaded and iron-loaded mice (Figure 1A). We also observed a significant reduction in nonheme liver iron content in both non-iron-loaded and iron-loaded pregnant mice at E12.5 mice when compared to nonpregnant controls (Figure 1B). For both groups, liver iron content was reduced further at E18.5, at which point it was significantly lower than that of E12.5 mice and nonpregnant animals. In response to the decrease in liver iron levels, expression of the hepcidin regulator bone morphogenetic protein 6 (*Bmp6*) [21,22] was also reduced in the liver of non-iron-loaded and iron-loaded pregnant mice at both E12.5 and E18.5 when compared to nonpregnant animals (Figure 1C). Likewise, the hepatic expression of SMAD family member 7 (*Smad7*), a downstream target of BMP signaling was also decreased at both E12.5 and E18.5 when compared to nonpregnant mice, although the decrease in non-iron-loaded pregnant mice at E18.5 did not reach statistical significance (Figure 1D). Serum iron and transferrin saturation were reduced at E12.5 and E18.5 in both non-iron-loaded and iron-loaded pregnant animals when compared to nonpregnant mice (Figure 1E,F). These data demonstrate that, by mid-pregnancy, liver iron stores and transferrin saturation, both of which are known regulators of hepcidin production, are decreased when compared to their respective controls in non-iron-loaded and iron-loaded animals.

### 2.2. Erythropoietic Activity Is Increased in the Spleen and Bone Marrow of Pregnant Mice by E12.5

**Figure 2:**
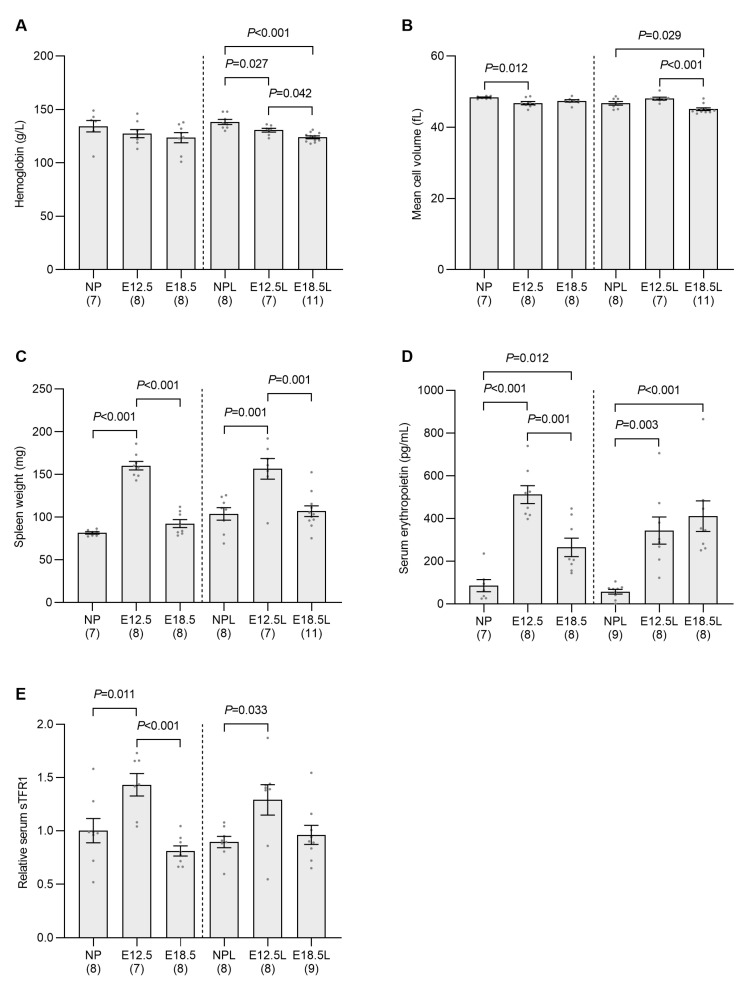
Erythropoietic activity is increased in pregnant mice by embryonic day 12.5. Non-iron-loaded and iron-loaded pregnant mice were examined at E12.5 or E18.5 and compared to age-matched nonpregnant mice to investigate the effect of pregnancy progression on hemoglobin (**A**), mean corpuscular volume (**B**), spleen weight (**C**), serum erythropoietin (**D**) and sTFR1 levels (**E**). Data are expressed as mean ± SEM with the number of mice in each group indicated in parentheses along the *x*-axis. Statistically significant differences between groups were determined using one-way ANOVA followed by either Tukey or Games–Howell post-hoc testing, with *p* values indicated for each significantly different comparison. NP: nonpregnant mice; E12.5: pregnant mice studied at embryonic day 12.5; E18.5: pregnant mice studied at embryonic day 18.5; NPL: iron-loaded nonpregnant mice; E12.5L: iron-loaded pregnant mice studied at embryonic day 12.5; E18.5L: iron-loaded pregnant mice studied at embryonic day 18.5.

Iron demand from the developing fetus is highest in the latter part of pregnancy, and so a decrease in maternal iron status would be expected at this time. However, we also show a significant decrease in markers of maternal iron status at E12.5, indicating an increase in iron utilization at this early stage of pregnancy, when fetal iron demand is expected to be relatively low. To determine the cause of this early elevation in iron demand, we examined erythropoiesis in our pregnant animals, as most of the circulating iron in nonpregnant mice is utilized for erythrocyte development. Maternal hemoglobin levels were unchanged across gestation in non-iron-loaded pregnant animals; however, small but significant decreases in hemoglobin were seen in iron-loaded pregnant mice at E12.5 and E18.5 when compared to nonpregnant controls (Figure 2A). Small decreases in mean cell volume were also seen at E12.5 in non-iron-loaded pregnant mice and E18.5 in iron-loaded pregnant animals when compared to their respective nonpregnant controls (Figure 2B).

The observation that only slight decreases in hematological parameters were recorded at E18.5, when plasma volumes have increased by approximately 80% by this stage of pregnancy [23], demonstrates that significant erythropoiesis occurred during pregnancy. As erythropoiesis is a very iron-demanding process, this implies that there was a significant increase in maternal iron utilization prior to E18.5 independent of fetal iron requirements. Further examination of maternal physiology revealed a doubling of spleen weight at E12.5 in non-iron-loaded pregnant mice and a 50% increase in iron-loaded pregnant animals when compared to their respective nonpregnant controls (Figure 2C). In both models, spleen weight decreased to control levels by E18.5. Enlargement of the spleen often signifies an increase in erythropoiesis [24]. To examine whether there was an increase in erythropoietic drive at E12.5, we measured the level of erythropoietin in the serum and found it to be increased sixfold in both non-iron-loaded and iron-loaded pregnant mice at E12.5 when compared to nonpregnant controls (Figure 2D). Erythropoietin levels had reduced by E18.5 in non-iron-loaded mice but remained elevated when compared to nonpregnant animals. Serum soluble transferrin receptor 1 (sTFR1), a marker of erythroid iron demand, was also elevated at E12.5 in both non-iron-loaded and iron-loaded pregnant animals when compared to their respective controls (Figure 2E).

**Figure 3:**
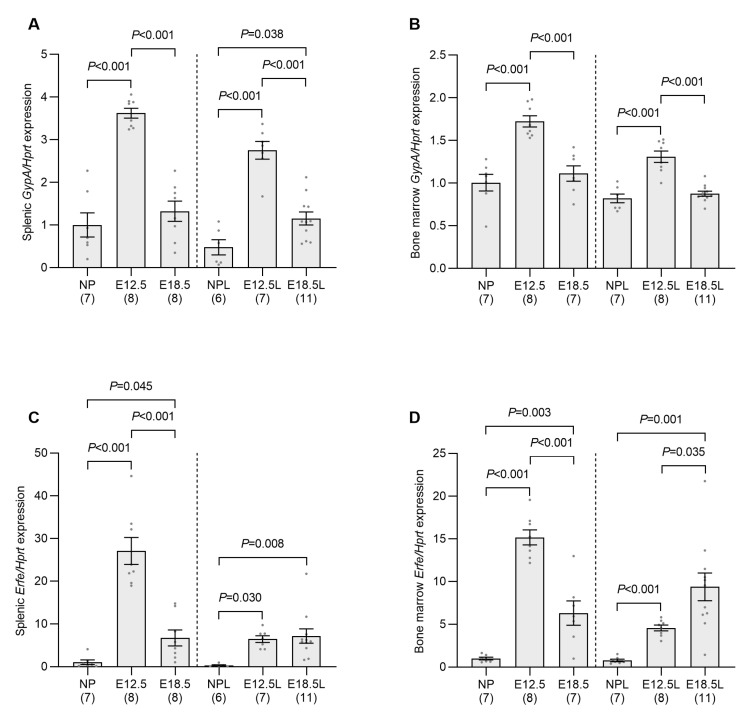
Increased erythropoiesis at E12.5 correlates with *GypA* and *Erfe* mRNA expression in the spleen and bone marrow. Non-iron-loaded and iron-loaded pregnant mice were examined at E12.5 or E18.5 and compared to age-matched nonpregnant mice to investigate the effect of pregnancy progression on the expression of the erythrocyte marker *GypA* in the spleen (**A**) and bone marrow (**B**), the hepcidin suppressor *Erfe* in the spleen (**C**) and bone marrow (**D**). Gene expression was calculated relative to the housekeeping gene *Hprt* and is expressed as a proportion of levels in the nonpregnant group. Data are expressed as mean ± SEM with the number of mice in each group indicated in parentheses along the *x*-axis. Statistically significant differences between groups were determined using one-way ANOVA followed by either Tukey or Games–Howell post-hoc testing, with *p* values indicated for each significantly different comparison. NP: nonpregnant mice; E12.5: pregnant mice studied at embryonic day 12.5; E18.5: pregnant mice studied at embryonic day 18.5; NPL: iron-loaded nonpregnant mice; E12.5L: iron-loaded pregnant mice studied at embryonic day 12.5; E18.5L: iron-loaded pregnant mice studied at embryonic day 18.5.

We observed a significant increase in glycophorin A (*GypA*) in the spleen and bone marrow at E12.5 when compared to nonpregnant controls (Figure 3A,B), indicating an increase in red blood cell precursors in these tissues. *GypA* expression had returned to control levels by E18.5 in all instances apart from in spleens from iron-loaded pregnant mice, which remained slightly elevated when compared to nonpregnant animals, but below that of E12.5 mice. The expression of erythroferrone (*Erfe*)—an inhibitor of hepcidin produced by developing erythrocytes [25]—in the bone marrow and spleen was also significantly increased at E12.5 when compared to nonpregnant controls (Figure 3C,D), with expression in non-iron-loaded animals decreasing by E18.5 in both tissues, although not to the level of nonpregnant controls. In contrast, *Erfe* remained elevated at E18.5 in the spleen from iron loaded animals when compared to nonpregnant controls and increased further in bone marrow when compared to mice at E12.5. Attempts were made to measure serum ERFE concentration using a commercial ELISA; however, no significant changes were observed (Figure S1). As the manufacturer’s instructions accompanying the ELISA stated that ERFE levels in normal C57BL/6 mice are below the detection limit of the assay, it is unclear whether serum EFRE was unchanged in our pregnant animals or if the assay was not sensitive enough to detect any changes occurring. Regardless of the serum ERFE results, these data collectively indicate that there is a significant increase in erythropoietic activity, a known suppressor of hepcidin, at mid-pregnancy in both non-iron-loaded and iron-loaded animals.

**Figure 4:**
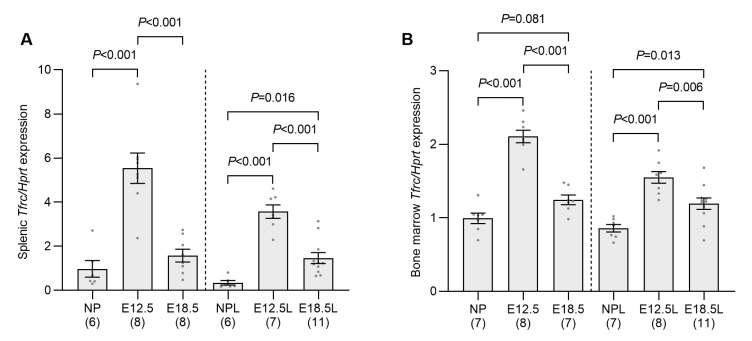
*Tfrc* expression is increased in the spleen and bone marrow at embryonic day 12.5 of pregnancy. Non-iron-loaded and iron-loaded pregnant mice were examined at E12.5 or E18.5 and compared to age-matched nonpregnant mice to investigate the effect of pregnancy progression on the expression of the iron uptake molecule *Tfrc* the spleen (**A**) and bone marrow (**B**). Gene expression was calculated relative to the housekeeping gene *Hprt* and is expressed as a proportion of levels in the nonpregnant group. Data are expressed as mean ± SEM with the number of mice in each group indicated in parentheses along the *x*-axis. Statistically significant differences between groups were determined using one-way ANOVA followed by either Tukey or Games–Howell post-hoc testing, with *p* values indicated for each significantly different comparison. NP: nonpregnant mice; E12.5: pregnant mice studied at embryonic day 12.5; E18.5: pregnant mice studied at embryonic day 18.5; NPL: iron-loaded nonpregnant mice; E12.5L,: iron-loaded pregnant mice studied at embryonic day 12.5; E18.5L: iron-loaded pregnant mice studied at embryonic day 18.5.

**Figure 5:**
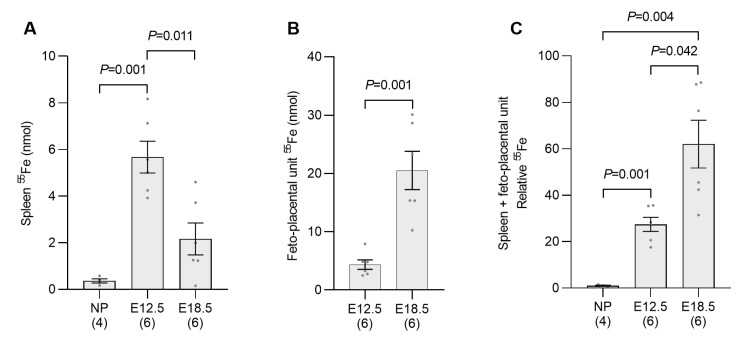
Iron uptake from the circulation is increased by embryonic day 12.5 of pregnancy. Non-iron-loaded pregnant mice were injected with ^55^Fe at E12.5 or E18.5 and euthanized 2 h later to examine iron uptake in the entire spleen (**A**) and fetoplacental unit (**B**). Absolute values from the spleen and fetoplacental unit were then combined to produce a graph of the total uptake from these two organ systems (**C**) represented as a proportion of the amount of ^55^Fe taken up by the spleen of nonpregnant mice. Data are expressed as mean ± SEM with the number of mice in each group indicated in parentheses along the *x*-axis. Statistically significant differences between groups were determined using one-way ANOVA followed by either Tukey or Games–Howell post-hoc testing, with *p* values indicated for each significantly different comparison. NP: nonpregnant mice; E12.5: pregnant mice studied at embryonic day 12.5; E18.5: pregnant mice studied at embryonic day 18.5.

In addition to the production of ERFE, the increased iron demands of stimulated erythropoiesis can also inhibit hepcidin production by reducing the amount of iron bound to transferrin in the circulation [26,27]. The elevated iron uptake capacity of the bone marrow and spleen at E12.5 of pregnancy was indicated by the increased expression of transferrin receptor 1 (*Tfrc*) mRNA (Figure 4A,B), the protein product of which is essential for iron uptake during erythrocyte development [28]. *Tfrc* expression decreased by E18.5 in both bone marrow and spleen from non-iron-loaded pregnant mice; however, it remained elevated above nonpregnant levels in iron-loaded animals. To directly measure changes in iron uptake, mice were injected with ^55^Fe, a radioactive isotope of iron, and its distribution was determined 2 h later. The uptake of ^55^Fe by the entire spleen had increased by more than 15-fold at E12.5 when compared to nonpregnant controls (Figure 5A). The ^55^Fe taken up by the fetoplacental unit (the ^55^Fe in all placentas and fetuses from a single mother) at E12.5 was surprisingly high, with the total ^55^Fe taken up equating to 75% of spleen uptake at this timepoint (spleen—5.7 nmol; fetoplacental unit—4.3 nmol) and double that of the spleen at E18.5 (2.2 nmol) (Figure 5B). Unsurprisingly, ^55^Fe uptake by the fetoplacental unit was almost fivefold greater at E18.5 than E12.5 (Figure 5B). The total counts from the spleen and the fetoplacental unit at each timepoint were then combined to determine the total ^55^Fe taken up by these two organs at E12.5 and E18.5 and these values compared to the total splenic uptake in nonpregnant mice. This analysis showed that the total amount of iron taken up from the circulation by the spleen and fetoplacental unit increased by 27-fold at E12.5 and 62-fold at E18.5 when compared to the level of uptake by the spleen alone in nonpregnant mice (Figure 5C). This clearly demonstrates that the amount of iron being removed from the circulation is significantly higher at E12.5 and E18.5 of pregnancy than it is in nonpregnant mice.

### 2.3. Increasing Maternal Iron Status Overcomes the Suppression of Maternal Hamp Expression at E18.5

**Figure 6:**
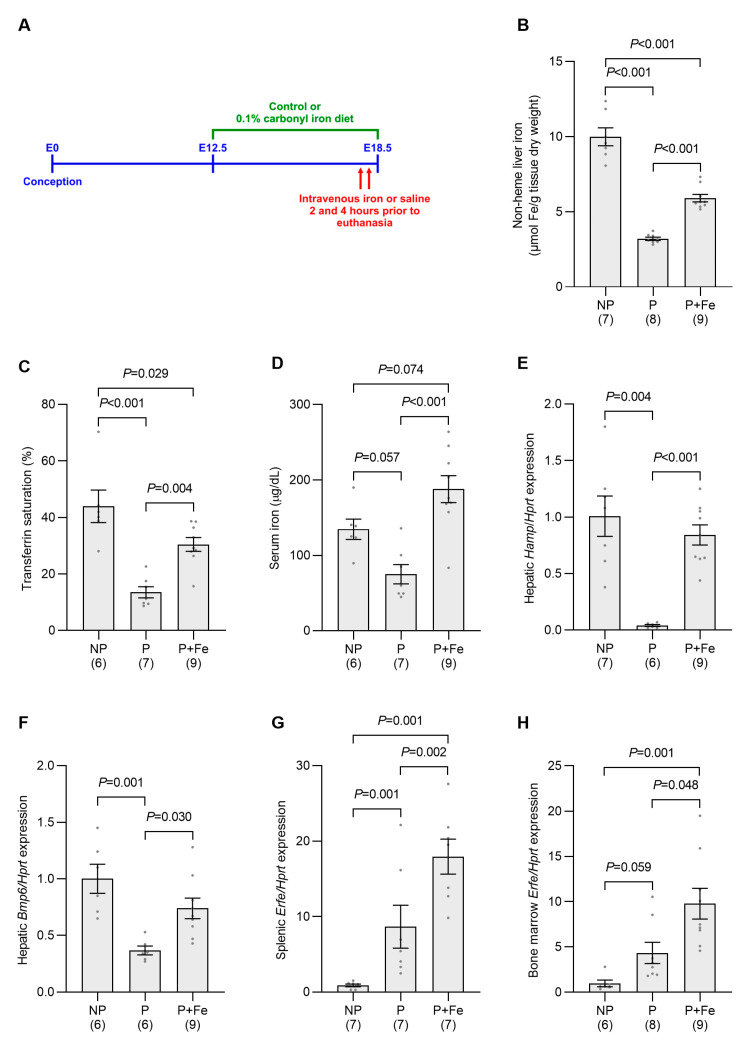
Hepatic *Hamp* expression is induced in pregnant mice treated with iron. Hepatic iron stores in pregnant mice were increased by switching to a 0.1% carbonyl iron diet from E12.5 and circulating iron was elevated by intravenous iron at 2 and 4 h prior to euthanasia at E18.5 (**A**), as described in Methods. Control nonpregnant and pregnant mice were switched to the control diet and injected with saline only. Hepatic nonheme iron content (**B**), transferrin saturation (**C**), serum iron levels (**D**), the expression of hepatic *Hamp* (**E**) and *Bmp6* (**F**) and *Erfe* expression in the bone marrow (**G**) and spleen (**H**) were then determined for each animal. Gene expression was calculated relative to the housekeeping gene *Hprt* and is expressed as a proportion of levels in the nonpregnant group. Data are expressed as mean ± SEM with the number of mice in each group indicated in parentheses along the *x*-axis. Statistically significant differences between groups were determined using one-way ANOVA followed by either Tukey or Games–Howell post-hoc testing, with *p* values indicated for each significantly different comparison. NP: nonpregnant control mice; P: pregnant control mice; P + Fe: pregnant mice treated with iron.

As both liver iron levels and transferrin saturation, which are known regulators of *Hamp* expression [13,29], are decreased in our pregnancy model, we next determined the effect of increasing these parameters on hepatic *Hamp* at E18.5. Liver iron levels were increased by switching pregnant non-iron-loaded mice to a 0.1% carbonyl iron-containing diet at E12.5 and transferrin saturation was elevated using intravenous iron injections as described in Materials and Methods (Figure 6A). These manipulations significantly increased liver iron concentration, transferrin saturation and serum iron levels when compared to pregnant mice not treated with iron (Figure 6B–D). Despite the levels of most iron parameters remaining lower than those of nonpregnant controls, iron treatment returned hepatic *Hamp* mRNA to a level that was not statistically different to the nonpregnant group (Figure 6E). These results suggest that the main driver of hepcidin suppression in pregnancy at E18.5 is the reduction in maternal iron levels.

An examination of known regulators of the *Hamp* signaling pathway was then undertaken. While pregnancy reduced hepatic *Bmp6* expression compared with nonpregnant mice, iron treatment returned *Bmp6* to a level not significantly different from that of nonpregnant controls, but significantly elevated when compared to pregnant mice not treated with iron (Figure 6F). *Erfe* expression increased approximately twofold in both the bone marrow and the spleen in response to iron manipulation when compared to untreated pregnant mice (Figure 6G,H).

**Figure 7:**
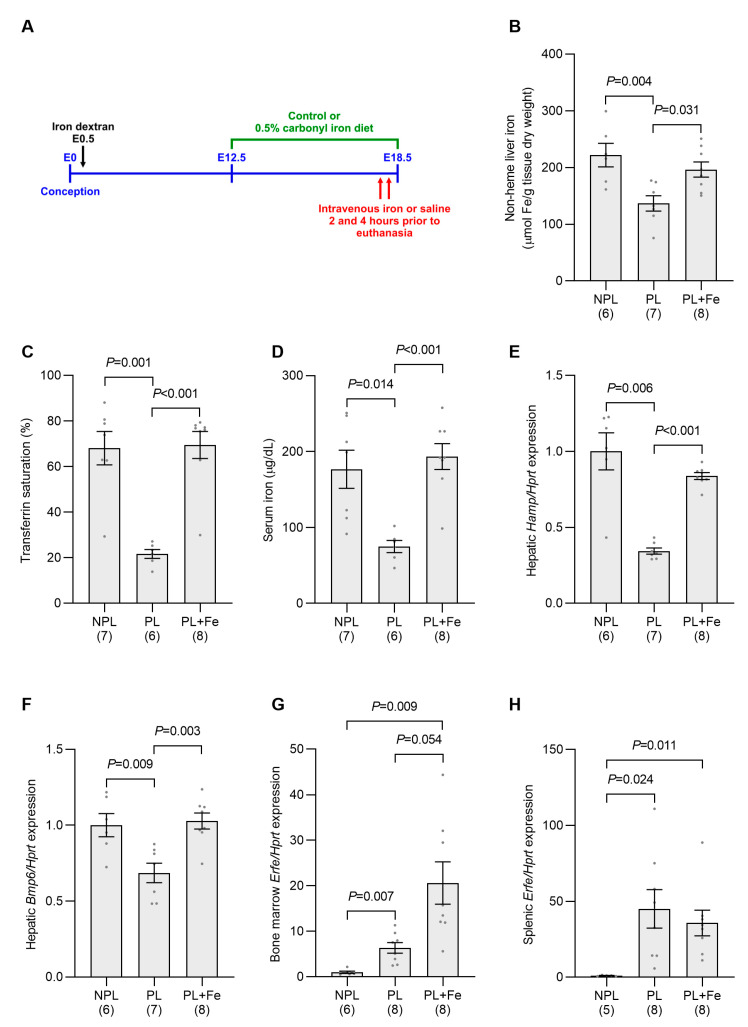
Increasing maternal iron status in iron-loaded pregnant mice induces hepatic *Hamp* expression. Pregnant mice were iron-loaded by injection with iron dextran at E0.5. Hepatic iron stores in iron-loaded pregnant mice were increased by switching to a 0.5% carbonyl iron diet from E12.5 and circulating iron was elevated by intravenous iron at 2 and 4 h prior to euthanasia at E18.5 (**A**), as described in Methods. Control nonpregnant and pregnant mice were switched to the control diet and injected with saline only. Hepatic nonheme iron content (**B**), transferrin saturation (**C**), serum iron levels (**D**), the expression of hepatic *Hamp* (**E**) and *Bmp6* (**F**) and *Erfe* expression in the bone marrow (**G**) and spleen (**H**) were then determined for each animal. Gene expression was calculated relative to the housekeeping gene *Hprt* and is expressed as a proportion of levels in the nonpregnant group. Data are expressed as mean ± SEM with the number of mice in each group indicated in parentheses along the *x*-axis. Statistically significant differences between groups were determined using one-way ANOVA followed by either Tukey or Games–Howell post-hoc testing, with *p* values indicated for each significantly different comparison. NPL: nonpregnant iron-loaded control mice; PL: pregnant iron-loaded control mice; PL + Fe: pregnant iron-loaded mice treated with iron.

Similar responses to iron treatment were seen in pregnant mice loaded with iron by injection with iron dextran at E0.5. Hepatic iron stores were maintained at high levels by switching the mice to a 0.5% carbonyl iron diet at E12.5 and the level of circulating iron was increased by intravenous iron injections prior to euthanasia on E18.5 (Figure 7A). These treatments led to significant increases in hepatic iron, transferrin saturation and serum iron levels when compared with untreated pregnant mice (Figure 7B–D). As a result of altering maternal iron status, hepatic *Hamp* expression was significantly increased when compared to untreated pregnant animals (Figure 7E). These data suggest that, as in normal pregnancy, changes in maternal iron status are the predominant regulators of hepcidin in iron-loaded pregnancy.

As with non-iron-loaded pregnancy, iron treatment of iron-loaded pregnant mice increased *Bmp6* expression when compared to untreated iron-loaded pregnant mice (Figure 7F). While *Erfe* expression remained elevated in the bone marrow and spleen from pregnant mice treated with iron when compared to iron-loaded nonpregnant mice, expression levels were not statistically different from those of untreated pregnant animals (Figure 7G,H). No change in the number of fetuses per pregnancy was evident between the iron-treated group and untreated mice in any of the experiments shown in Figure 6 and Figure 7 (Figure S2).

## 3. Discussion

In the current study, we examined the effect of maternal iron status on hepcidin production during pregnancy. Consistent with previous studies, we found that *Hamp* expression is significantly suppressed in late gestation [16,17]. This correlates with a decrease in hepatic iron stores and transferrin saturation, both known regulators of hepcidin production, implying an increase in iron demand at this time. Indeed, a previous study showed a 50% increase in plasma iron turnover in pregnant mice on days 17–18 of gestation compared with day 5 of pregnancy [30], and we show that this is largely attributable to increased iron uptake by the developing fetuses. We also show that increasing maternal iron status in pregnant mice simulates hepatic *Hamp* expression in both non-iron-loaded and iron-loaded pregnant mice. These results suggest that decreases in maternal hepatic and serum iron levels due to increased fetal iron demands in late gestation are the likely cause of hepcidin suppression at this time.

While fetal iron requirements are high in late gestation, demand is much lower mid-pregnancy due to the smaller size of the placenta and embryos. However, we found a significant decrease in *Hamp* expression at E12.5, which is consistent with previous findings [16]. This also coincided with decreases in serum iron, transferrin saturation and liver iron content, suggesting an increase in maternal iron utilization. Interestingly, we observed a doubling of spleen weight at E12.5 compared to nonpregnant control mice. This agrees with previous studies showing that, during pregnancy, spleen weight increases, reaching its peak mid-gestation [24,31,32]. Spleen weight in mice often increases in response to stress erythropoiesis [24,33] and, in the current study, we found higher levels of circulating erythropoietin, and elevated markers of erythropoiesis in the spleen and bone marrow, most of which had decreased to normal or near-normal levels by late-gestation. Previous studies have also shown an increase in splenic red pulp and erythroid progenitors [31], and enhanced hemoglobin staining in bone marrow and spleen [24] at mid-pregnancy in mice, indicating increased erythropoiesis. Furthermore, we found a 15-fold increase in the uptake of iron by the spleen at E12.5 and, when combined with uptake from the fetoplacental unit, a 27-fold increase in iron utilization at this stage of pregnancy. As an increase in erythropoietic iron demand is a strong inhibitor of hepcidin production [34], it is, therefore, not surprising that we see a decrease in *Hamp* expression at this time. Overall, these studies imply that maternal iron status is the major regulator of maternal hepcidin production during pregnancy and that, if there are any yet-to-be discovered pregnancy factors, their effects are likely minimal.

Interestingly, we found an increase in the expression of *Erfe* mRNA in the spleen and bone marrow of our E12.5 mothers; however, the levels of circulating ERFE were not significantly higher than in nonpregnant mice. This does not exclude a role for ERFE in pregnancy, as circulating levels are normally very low, with accurate detection using ELISA only possible in conditions of extreme erythroid stimulation, such as in β-thalassemia [35]. A study showing that pregnant mice lacking ERFE produce smaller erythrocytes—consistent with iron deficiency—supports a role for this hormone in the maintenance of erythroid iron supply during pregnancy [36]. ERFE suppresses hepcidin by binding to BMP6 and preventing the activation of the SMAD signaling pathway [37]; however, the level required for suppression likely depends on the amount of BMP6 present. The iron loading that occurs in β-thalassemia leads to high levels of BMP6 [33] and so more ERFE would presumably be required to suppress BMP6 activity. In contrast, *Bmp6* expression was reduced in our study at E12.5, suggesting that less ERFE would be needed to inhibit BMP6 activity. Therefore, the enhanced ERFE production at E12.5, as indicated by the increase in *Erfe* mRNA in bone marrow and spleen, may be sufficient to further suppress BMP6 signaling. Other factors associated with increased erythropoiesis may also be acting to decrease *Hamp* expression during pregnancy independent of ERFE. The decrease in serum iron levels associated with stimulated erythropoiesis is one such factor [26,27]. In addition, β-thalassemic mice lacking ERFE develop significant iron loading, suggesting that unknown erythroid regulators of hepcidin expression remain to be identified [25,35].

The current study suggests that a specific pregnancy factor regulating hepcidin expression independent of iron levels is unlikely to exist or, if there is such a factor, it plays only a minor role during pregnancy. Our current understanding of hepcidin regulation also argues against such a factor. Hepcidin maintains iron homeostasis by regulating dietary iron absorption and the release of storage iron from hepatocytes and macrophages [10]. Hepcidin expression can be altered in two distinct scenarios. In the first scenario, changes in iron status alter hepcidin production in an attempt to return iron levels to normal. An example of this would be the decrease in hepcidin that occurs in iron deficiency. In the second scenario, hepcidin is altered independent of iron status in order to alter iron parameters away from normal. An example is acute inflammation, where an increase in hepcidin leads to a rapid reduction in blood iron levels [38]. Thus, hepcidin levels are either altered by changes in iron-dependent parameters or are altered by iron-independent factors so that iron parameters are changed. It is not possible for an iron-independent factor to alter hepcidin levels without also causing a change in iron status, as this could only be achieved if hepcidin has no effect on iron homeostasis. Therefore, a hypothetical pregnancy factor that reduces hepcidin in an iron-independent manner, i.e., prior to any change in iron status, should result in a rapid increase in iron parameters, such as serum iron levels, as extra iron is released into the circulation. An alternative possibility is that the effects of such a pregnancy factor on iron parameters are masked by the increased iron demands of pregnancy, i.e., the increase in iron entering the circulation is countered by an increase in iron utilization. However, there is no need to propose such a scenario, as an increase in iron utilization would decrease serum iron levels, thereby triggering the decrease in hepcidin seen. In fact, we would argue that there is no scenario identified thus far involving a reduction in hepcidin expression during pregnancy that cannot be adequately explained by changes in the known hepcidin regulatory factors related to hepatic iron stores, circulating iron levels or stimulated erythropoiesis. Therefore, not only is there no evidence supporting an iron-independent, pregnancy-specific hepcidin regulator, there is no biological need for one.

In conclusion, we demonstrated that increased iron requirements resulting in lower iron stores and transferrin saturation are the most likely signals suppressing hepcidin during pregnancy. Our data suggest that there are two significant events that increase iron demand in pregnant mice. Firstly, elevated erythropoiesis and the development of the placenta results in increased iron utilization by mid-gestation, which decreases transferrin saturation, suppressing hepcidin production. The reduction in circulating hepcidin releases iron from storage sites such as the liver, causing the decrease in hepatic iron seen and further reducing hepcidin. Secondly, while erythropoietic activity returns to near-normal levels as pregnancy progresses, the increasing iron demands of multiple fetuses keeps transferrin saturation low and exacerbates the reduction in hepatic iron stores, further suppressing hepcidin. As the repression of hepcidin can be explained by changes in known hepcidin regulators, there would appear to be no need for inhibition from a yet-to-be discovered, iron-independent pregnancy factor, and any involvement from such a factor would appear to be minimal.

## 4. Materials and Methods

### 4.1. Animals

Nulliparous female C57BL/6 mice were maintained on a standard rodent pellet diet (Rat and Mouse Nuts; 120 mg/kg iron Norco Stockfeed, Lismore, Australia) and time-mated at 8–12 weeks of age. To iron load mice, animals were administered an intraperitoneal injection containing 300 μg iron as iron dextran per gram body weight on embryonic day 0.5 (E0.5), as has been described previously [16]. Pregnant mice were euthanized at E12.5 or E18.5. Mice that did not become pregnant were used as nonpregnant controls. Prior to euthanasia, mice were anesthetized (200 mg/kg ketamine, 10 mg/kg xylazine) and blood was withdrawn by cardiac puncture. Blood was aliquoted into either EDTA tubes for hematological analysis or allowed to clot and the serum collected and stored at −80 °C for serum iron analysis. Following euthanasia, maternal liver, bone marrow and spleen were collected and snap frozen for subsequent analysis.

To examine the effect of manipulating maternal iron levels on hepcidin production, mice were treated in the following manner. Liver iron stores were increased in pregnant mice by switching animals from the standard rodent pellet diet to a diet based on AIN-93G (Specialty Feeds, Glen Forrest, Australia) containing 0.1% carbonyl iron at E12.5. Serum iron levels were increased by intravenous injection (2.5 µg/g body weight ferric citrate monohydrate in 5 mM citrate buffer, pH 7.0) at both 2 and 4 h prior to euthanasia. Two doses were required to maintain a high level of iron in the circulation due to the rapid utilization of iron in late pregnancy. Control animals were switched to the AIN-93G-based diet containing 50 mg/kg iron as ferric citrate and injected as described above with 5 mM citrate buffer, pH 7.0. Pregnant mice that had been injected with iron dextran at E0.5 were treated similarly except that the diet contained 0.5% carbonyl iron as this was required to maintain iron stores at high levels.

### 4.2. Tissue Uptake of Radioactive Iron

Pregnant mice at either E12.5 or E18.5 or nonpregnant controls were injected intravenously with 10 µCi of ^55^Fe (Perkin Elmer, Glen Waverley, Australia) in 5 mM citrate buffer, pH 7.0 and euthanized 2 h later. Whole mouse tissues were liquefied in 5 mL Soluene-350 (Perkin Elmer) overnight in a shaking incubator at 70°. Following liquification, H_2_O_2_ was added (500 µL for whole fetuses, 200 µL for placenta and 100 µL for maternal spleen) and each sample incubated at 70 °C with shaking for 2 h to minimize color quenching. A portion (1 mL) of each liquefied sample was then added to 10 mL Hionic-Fluor Scintillation Cocktail (Perkin Elmer) and the ^55^Fe in each sample counted using a TRICARB-4910TR Scintillation Counter (Perkin Elmer). Any residual chemical quenching in each sample was corrected for by using separate quench curves that were established for each tissue. A standard curve using known amounts of ^55^Fe was used to calculate the absolute amount of ^55^Fe in each tissue.

### 4.3. Hematological Parameters and Iron Status Markers

Hematological parameters were measured using a Coulter Ac·T diff Hematology Analyzer (Beckman Coulter, Gladesville, Australia). Total serum iron and transferrin saturation were measured using the Iron/TIBC Reagent Set (Pointe Scientific, Canton, MI, USA) as previously described [39]. Maternal liver nonheme iron was determined using a colorimetric assay [40].

### 4.4. Serum Erythropoietin and sTFR

Serum erythropoietin was determined using a commercial ELISA (intra-assay %CV—3–4.4%; inter-assay %CV—2.5–9.7%; detection limit—18.0 pg/mL; MEP00B: In Vitro Technologies, Noble Park, Australia) according to the manufacturer’s instructions. Serum erythroferrone levels were determined using a commercial ELISA (intra-assay %CV—4.7–6.7%; inter-assay %CV—7.0–14.9%; detection limit—0.16 ng/mL; ERF-001, Intrinsic Lifesciences, La Jolla, CA, USA) as per the manufacturer’s instructions. Serum sTFR levels were determined using ELISA as previously described [40,41] and as briefly outlined here. All incubations were performed at room temperature with constant shaking unless otherwise indicated. Phosphate-buffered saline plus 0.05% Tween-20 was used for all wash steps. Ninety-six-well ELISA plates were first coated with rat antimouse CD71 (SouthernBiotech, Birmingham, AL, USA). Each well was then washed before being incubated with blocking buffer (5% skim milk powder in phosphate buffered saline plus 0.05% Tween-20) for one hour. Following washing, serum samples were added to each well (diluted one in 50 with blocking buffer) and the plate incubated for one hour. Wells were then washed before 0.25 μg/mL biotin labelled rat antimouse CD71 (BD, North Ryde, Australia) in blocking buffer was added and the plate incubated for one hour. Following washing, streptavidin-HRP (1:8000, Cell Signaling Technology, Danvers, MA, USA) was added and the plate incubated for one hour. Each well was then washed, TMB Substrate Solution (Cell Signaling Technology) was added and the color allowed to develop for 15 min at room temperature without shaking. Stop Solution (Cell Signaling Technology) was then added and the absorbance of each well, measured at 450 nm. The relative expression of sTFR in each sample was determined from a standard curve produced from *Hbb*^th3/+^ mice, which have elevated levels of sTFR [40]. The intra-assay %CV for this assay is 2.4–6.5% and the inter-assay %CV is 3.5–8%.

### 4.5. RNA Extraction and Gene Expression Analysis

RNA was extracted from liver, spleen and bone marrow using TRIzol reagent (Thermo Fisher Scientific, Seventeen Mile Rocks, Australia) as per the manufacturer’s instructions. cDNA was synthesized from spleen and bone marrow RNA (2000 ng) using Superscript III reverse transcriptase (Thermo Fisher Scientific) or from liver RNA (800 ng) using Moloney Murine Leukemia Virus Reverse Transcriptase (Thermo Fisher Scientific), both with an oligo(dT) primer according to the manufacturer’s instructions. Real-time qPCR to assess gene expression was performed using iTaq Universal SYBR Green Supermix (Bio-Rad, Gladesville, Australia) and a CFX384 Touch Real-Time PCR Detection System (Bio-Rad). Gene expression was calculated from the threshold cycle value using a standard curve and was expressed relative to the housekeeping gene hypoxanthine guanine phosphoribosyl transferase (*Hprt*). Gene-specific primers are listed in Table 1, and the validation of primers and subsequent analysis is consistent with the Minimum Information for Publication of Quantitative Real-Time PCR Experiments guidelines [42].

### 4.6. Statistical Analysis

The results are expressed as mean ± SEM. Statistically significant differences between groups were calculated with SPSS Statistics version 23 software (IBM Australia, St Leonards, Australia), using one way ANOVA followed by either Tukey post-hoc testing for samples with equal variance or Games–Howell post-hoc testing for samples of unequal variance. The equality of the variances between groups was determined using Levene’s test. A *p* value less than 0.05 was considered statistically significant.

## Figures and Tables

**Table 1:** qPCR primers.

Gene	Forward	Reverse
*Bmp6*	AACAGCTTGCAAGAAGCATGAG	TGGACCAAGGTCTGTACAATGG
*Erfe*	CCAGGCCCCTTTATCCCATC	GTGCTCCAGATGGCTCTCTC
*GypA*	GTGATGGCAGGGATTATCGGA	CACTGTTGTCACCACCCTCA
*Hamp*	CCTGAGCAGCACCACCTATC	TGCAACAGATACCACACTGGG
*Hprt*	ATGATCAGTCAACGGGGGAC	TTGGGGCTGTACTGCTTAAC
*Smad7*	GGCATTCCTCGGAAGTCAAG	CAGCCTGCAGTTGGTTTGAG
*Tfrc*	TCATGAGGGAAATCAATGATCG	CCAGAGCAGCTTAAATCC

## Data Availability

The datasets used and/or analyzed during the current study are available from the corresponding author on reasonable request.

## Supplementary Materials

The following supporting information can be downloaded at: https://www.mdpi.com/article/10.3390/ijms241814379/s1.

## References

[1] Breymann C. (2015). Iron Deficiency Anemia in Pregnancy. Semin. Hematol..

[2] Lozoff B., Georgieff M.K. (2006). Iron deficiency and brain development. Semin. Pediatr. Neurol..

[3] Rao R., Georgieff M.K. (2007). Iron in fetal and neonatal nutrition. Semin. Fetal Neonatal. Med..

[4] Kulik-Rechberger B., Kosciesza A., Szponar E., Domosud J. (2016). Hepcidin and iron status in pregnant women and full-term newborns in first days of life. Ginekol. Pol..

[5] van Santen S., Kroot J.J., Zijderveld G., Wiegerinck E.T., Spaanderman M.E., Swinkels D.W. (2013). The iron regulatory hormone hepcidin is decreased in pregnancy: A prospective longitudinal study. Clin. Chem. Lab. Med..

[6] Pigeon C., Ilyin G., Courselaud B., Leroyer P., Turlin B., Brissot P., Loreal O. (2001). A new mouse liver-specific gene, encoding a protein homologous to human antimicrobial peptide hepcidin, is overexpressed during iron overload. J. Biol. Chem..

[7] Abboud S., Haile D.J. (2000). A novel mammalian iron-regulated protein involved in intracellular iron metabolism. J. Biol. Chem..

[8] McKie A.T., Marciani P., Rolfs A., Brennan K., Wehr K., Barrow D., Miret S., Bomford A., Peters T.J., Farzaneh F. (2000). A novel duodenal iron-regulated transporter, IREG1, implicated in the basolateral transfer of iron to the circulation. Mol. Cell..

[9] Donovan A., Brownlie A., Zhou Y., Shepard J., Pratt S.J., Moynihan J., Paw B.H., Drejer A., Barut B., Zapata A. (2000). Positional cloning of zebrafish ferroportin1 identifies a conserved vertebrate iron exporter. Nature.

[10] Nemeth E., Tuttle M.S., Powelson J., Vaughn M.B., Donovan A., Ward D.M., Ganz T., Kaplan J. (2004). Hepcidin regulates cellular iron efflux by binding to ferroportin and inducing its internalization. Science.

[11] Anderson G.J., Frazer D.M. (2017). Current understanding of iron homeostasis. Am. J. Clin. Nutr..

[12] Nicolas G., Chauvet C., Viatte L., Danan J.L., Bigard X., Devaux I., Beaumont C., Kahn A., Vaulont S. (2002). The gene encoding the iron regulatory peptide hepcidin is regulated by anemia, hypoxia, and inflammation. J. Clin. Investig..

[13] Frazer D.M., Anderson G.J., Collins J.F. (2017). Hepcidin and the hormonal control of iron homeostasis. Molecular, Genetic, and Nutritional Aspects of Major and Trace Minerals.

[14] Beaton G.H. (1966). Some physiological adjustments relating to nutrition in pregnancy. Can. Med. Assoc. J..

[15] Bothwell T.H. (2000). Iron requirements in pregnancy and strategies to meet them. Am. J. Clin. Nutr..

[16] Sangkhae V., Fisher A.L., Wong S., Koenig M.D., Tussing-Humphreys L., Chu A., Lelic M., Ganz T., Nemeth E. (2020). Effects of maternal iron status on placental and fetal iron homeostasis. J. Clin. Investig..

[17] Sangkhae V., Fisher A.L., Chua K.J., Ruchala P., Ganz T., Nemeth E. (2020). Maternal hepcidin determines embryo iron homeostasis in mice. Blood.

[18] Koenig M.D., Tussing-Humphreys L., Day J., Cadwell B., Nemeth E. (2014). Hepcidin and iron homeostasis during pregnancy. Nutrients.

[19] Ganz T. (2020). The role of hepcidin in fetal iron homeostasis. Blood.

[20] Donker A.E., van der Staaij H., Swinkels D.W. (2021). The critical roles of iron during the journey from fetus to adolescent: Developmental aspects of iron homeostasis. Blood Rev..

[21] Babitt J.L., Huang F.W., Xia Y., Sidis Y., Andrews N.C., Lin H.Y. (2007). Modulation of bone morphogenetic protein signaling in vivo regulates systemic iron balance. J. Clin. Investig..

[22] Kautz L., Meynard D., Monnier A., Darnaud V., Bouvet R., Wang R.H., Deng C., Vaulont S., Mosser J., Coppin H. (2008). Iron regulates phosphorylation of Smad1/5/8 and gene expression of Bmp6, Smad7, Id1, and Atoh8 in the mouse liver. Blood.

[23] Fruhman G.J. (1968). Blood formation in the pregnant mouse. Blood.

[24] Fowler J.H., Nash D.J. (1968). Erythropoiesis in the spleen and bone marrow of the pregnant mouse. Dev. Biol..

[25] Kautz L., Jung G., Valore E.V., Rivella S., Nemeth E., Ganz T. (2014). Identification of erythroferrone as an erythroid regulator of iron metabolism. Nat. Genet..

[26] Mirciov C.S.G., Wilkins S.J., Hung G.C.C., Helman S.L., Anderson G.J., Frazer D.M. (2018). Circulating iron levels influence the regulation of hepcidin following stimulated erythropoiesis. Haematologica.

[27] Artuso I., Pettinato M., Nai A., Pagani A., Sardo U., Billore B., Lidonnici M.R., Bennett C., Mandelli G., Pasricha S.R. (2019). Transient decrease of serum iron after acute erythropoietin treatment contributes to hepcidin inhibition by ERFE in mice. Haematologica.

[28] Wang S., He X., Wu Q., Jiang L., Chen L., Yu Y., Zhang P., Huang X., Wang J., Ju Z. (2020). Transferrin receptor 1-mediated iron uptake plays an essential role in hematopoiesis. Haematologica.

[29] Detivaud L., Nemeth E., Boudjema K., Turlin B., Troadec M.B., Leroyer P., Ropert M., Jacquelinet S., Courselaud B., Ganz T. (2005). Hepcidin levels in humans are correlated with hepatic iron stores, hemoglobin levels, and hepatic function. Blood.

[30] Jepson J.H., Lowenstein L. (1968). Role of erythropoietin and placental lactogen in the control of erythropoiesis during pregnancy. Can. J. Physiol. Pharmacol..

[31] Bustamante J.J., Dai G., Soares M.J. (2008). Pregnancy and lactation modulate maternal splenic growth and development of the erythroid lineage in the rat and mouse. Reprod. Fertil. Dev..

[32] Maroni E.S., de Sousa M.A. (1973). The lymphoid organs during pregnancy in the mouse. A comparison between a syngeneic and an allogeneic mating. Clin. Exp. Immunol..

[33] Frazer D.M., Wilkins S.J., Darshan D., Badrick A.C., McLaren G.D., Anderson G.J. (2012). Stimulated erythropoiesis with secondary iron loading leads to a decrease in hepcidin despite an increase in bone morphogenetic protein 6 expression. Br. J. Haematol..

[34] Vokurka M., Krijt J., Sulc K., Necas E. (2006). Hepcidin mRNA levels in mouse liver respond to inhibition of erythropoiesis. Physiol. Res..

[35] Kautz L., Jung G., Du X., Gabayan V., Chapman J., Nasoff M., Nemeth E., Ganz T. (2015). Erythroferrone contributes to hepcidin suppression and iron overload in a mouse model of beta-thalassemia. Blood.

[36] Sangkhae V., Yu V., Coffey R., O’Brien K.O., Ganz T., Nemeth E. (2022). Erythroferrone contributes to iron mobilization for embryo erythropoiesis in iron-deficient mouse pregnancies. Am. J. Hematol..

[37] Arezes J., Foy N., McHugh K., Sawant A., Quinkert D., Terraube V., Brinth A., Tam M., LaVallie E.R., Taylor S. (2018). Erythroferrone inhibits the induction of hepcidin by BMP6. Blood.

[38] Ganz T., Nemeth E. (2015). Iron homeostasis in host defence and inflammation. Nat. Rev. Immunol..

[39] Frazer D.M., Wilkins S.J., Darshan D., Mirciov C.S.G., Dunn L.A., Anderson G.J. (2017). Ferroportin Is Essential for Iron Absorption During Suckling, But Is Hyporesponsive to the Regulatory Hormone Hepcidin. Cell. Mol. Gastroenterol. Hepatol..

[40] Mirciov C.S., Wilkins S.J., Dunn L.A., Anderson G.J., Frazer D.M. (2017). Characterization of Putative Erythroid Regulators of Hepcidin in Mouse Models of Anemia. PLoS ONE.

[41] Salojin K.V., Cabrera R.M., Sun W., Chang W.C., Lin C., Duncan L., Platt K.A., Read R., Vogel P., Liu Q. (2011). A mouse model of hereditary folate malabsorption: Deletion of the PCFT gene leads to systemic folate deficiency. Blood.

[42] Bustin S.A., Benes V., Garson J.A., Hellemans J., Huggett J., Kubista M., Mueller R., Nolan T., Pfaffl M.W., Shipley G.L. (2009). The MIQE guidelines: Minimum information for publication of quantitative real-time PCR experiments. Clin. Chem..

