# Supplementary material for: A Decrease in Maternal Iron Levels Is the Predominant Factor Suppressing Hepcidin during Pregnancy in Mice
Source: Int J Mol Sci. 2023 Sep 21;24(18):14379. doi: 10.3390/ijms241814379 (PMC10532249; doi:10.3390/ijms241814379)
Supplement: Supplementary file 1 [file ijms-24-14379-s001.zip › ijms-2587404-supplementary.pdf]

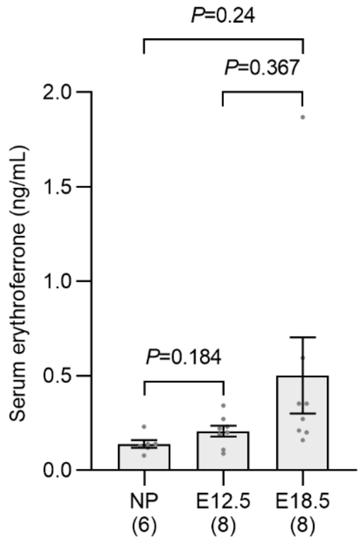

Figure S1. Serum ERFE levels in non-iron-loaded pregnant mice. **Non-iron-loaded** pregnant mice were examined at E12.5 or E18.5 and compared to age-matched nonpregnant mice to investigate the effect of pregnancy progression on serum ERFE levels. Data are expressed as mean ± SEM with the number of mice in each group indicated in parentheses along the x-axis. Statistically significant differences between groups were determined using one-way ANOVA followed by either Tukey or Games-Howell post-hoc testing, with *p* values indicated for each comparison. NP: nonpregnant mice; E12.5: pregnant mice studied at embryonic day 12.5; E18.5: pregnant mice studied at embryonic day 18.5.

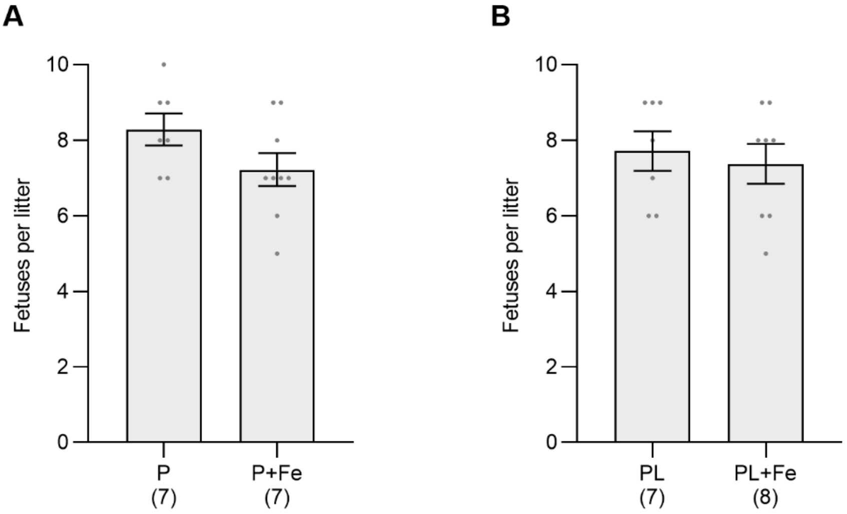

Figure S2. The number of fetuses in iron-treated pregnancies was not statistically different from untreated pregnant mice. Hepatic iron stores in pregnant mice were increased by switching to a 0.1% carbonyl iron diet from E12.5 and circulating iron was elevated by intravenous iron at 2 and 4 hours prior to euthanasia at E18.5. Iron-loaded pregnant mice were similarly treated using a 0.5% carbonyl iron diet. Control non-iron-loaded and iron-loaded pregnant mice were switched to the control diet and injected with saline only. The number of fetuses in non-iron-loaded (A) and iron-loaded (B) pregnant mice is shown. Data are expressed as mean ± SEM with the number of mice in each group indicated in parentheses along the x-axis. There were no significant difference between the groups. P: pregnant control mice; P + Fe: pregnant mice treated with iron; PL: pregnant iron-loaded control mice; PL + Fe, pregnant-iron loaded mice treated with iron.
